# The Early Clinical and Radiographic Outcomes of Robotic-Assisted Midline Lumbar Interbody Fusion (MIDLIF) With Expandable Interbody Spacers: A Case Series

**DOI:** 10.7759/cureus.82802

**Published:** 2025-04-22

**Authors:** Giada Garufi, Alfredo Conti, Domenicantonio Collufio, Francesco Messineo, Antonio Morabito, Giuseppe Ricciardo, Salvatore Cardali

**Affiliations:** 1 Neurosurgery, Papardo Hospital, University of Messina, Messina, ITA; 2 Neurosurgery, University of Bologna, Bologna, ITA; 3 Neurosurgery, Papardo Hospital, Messina, ITA

**Keywords:** cortical screws, expandable spacer, lumbar interbody fusion, robotics, robotic spine surgery

## Abstract

Objective: To assess the early clinical and radiological outcomes of the midline lumbar interbody fusion (MIDLIF) approach with the use of robotic assistance and expandable spacers.

Methods: A retrospective case series was performed on patients who underwent MIDLIF procedures for the treatment of degenerative spinal diseases, with a minimum of three months postoperative follow-up. Demographic (age, gender, body mass index [BMI], comorbidities, and diagnoses), surgical data (operative time, blood loss, hospital stay, intraoperative complications), patient-reported outcomes (PROs) (visual analogue scale [VAS] back pain and disability (Oswestry disability index [ODI]), and radiographic data were collected.

Results: In total, 42 patients were included, with an average age of 53.6 years and a BMI of 28 kg/m². Surgical data showed the mean total operative time was 98.8 minutes, and the mean blood loss was 17.1 mL with no intraoperative complications. At three-month follow-up, all mean PROs showed statistically significant improvement (p<0.05) when compared with baseline. VAS back pain improved from 7.5 (standard deviation (stdev) 7.5±0.7) preoperatively to 3.0 (stdev 1.0) at three months postoperative, while ODI improved from 58.7 (stdev 7.2) to 26.3 (stdev 10.3) at the same time points. Radiographic data showed that using robotic assistance for planning and placing cortical screws yielded high accuracy, as evidenced by a mean tip deviation of 1.2 mm (stdev 0.6 mm), mean tail deviation of 1.1 mm (stdev 0.4 mm) and a mean angular offset of 1.4 mm (stdev 0.7). Two (4.8%) patients had postoperative complications at three-month follow-up, including a wound infection and one report of wound dehiscence.

Conclusion: MIDLIF is an efficient, reproducible surgical procedure with a low complication rate that resulted in significant improvements in early PROs. Robotic assistance for planning and placing cortical screws in MIDLIF was highly accurate. These initial findings suggest that using robotic assistance adds value to MIDLIF procedures and is a viable alternative to traditional posterior fusion procedures.

## Introduction

Lumbar degenerative spine disease is a pervasive global issue that impacts individuals, medical systems, and society [1]. It is associated with low back pain and can significantly impact patients' quality of life. There are various strategies available to treat it, starting with conservative options, such as physical therapy, the use of NSAIDs, and targeted steroid injections. If conservative options are unsuccessful, surgical options are available. Conventional posterior approaches, which include transforaminal lumbar interbody fusion (TLIF) and posterior lumbar interbody fusion (PLIF), are commonly used surgical procedures for the treatment of these unresolved degenerative lumbar conditions [2]. They are well-established methods and have been used successfully for multiple decades. Despite their frequent use, they have some notable drawbacks, particularly with regard to complications [3-6].

Midline lumbar interbody fusion (MIDLIF) is a relatively new, mini-open surgical approach developed to address issues with the traditional PLIF and TLIF techniques [7-9]. In general, the MIDLIF approach is similar to PLIF but spares dissection of the paraspinal muscles through blunt dissection and utilizes a cortical bone trajectory for transpedicular fixation. Pedicle screws placed using a cortical bone trajectory have been shown to have a 30% increase in uniaxial yield pullout load compared to the traditional pedicle screws, particularly in patients with low bone mineral density, and have a low incidence of nerve root injury [9-11].

Currently, there has been an increase in the adoption of robotic assistance to place pedicle screws in spinal fusion surgeries. Robot assistance allows for pre-planning of the screw trajectory based on the patient's anatomy and helps guide screw placement through real-time navigation. Various papers have shown that robotic assistance has resulted in screw placement accuracy as high as 97-99% [12-14]. Based on this, the use of robot assistance in MIDLIF procedures is expected to provide accurate placement of pedicle screws placed using a cortical bone trajectory, which can be more nuanced to place than the traditional trajectory.

This study aims to assess the early clinical and radiographic outcomes achieved in patients who underwent MIDLIF and the accuracy of cortical screw placement with robotic assistance.

## Materials and methods

A retrospective review was undertaken of patients who underwent MIDLIF procedures performed by a single surgeon at Papardo Hospital, University of Messina, Sicily, from January 2023 to December 2023. The inclusion criteria for the study were that patients had to have 1) had placement of cortical screws with robotic assistance, 2) a minimum of three months of postoperative follow-up with evaluable clinical and complication data, 3) had placement of an expandable interbody spacer, 4) be 18 years of age or older, 5) be diagnosed with an on-label lumbar spinal condition, 6) not have been a trauma and/or tumor and/or pregnant patient.

Data collected from this study included demographics (age, gender, body mass index [BMI], comorbidities, and diagnoses), surgical data (operative time, estimated blood loss, hospital stay, and intraoperative complications), and patient-reported outcomes (PROs) (visual analog scale [VAS] back pain and disability (Oswestry disability index [ODI]). Radiographic measurements (computed tomography [CT] scans) were also taken to measure plan-to-place accuracy (screw tip deviation, screw tail deviation, and angular offset). The PROs and CT scans were recorded preoperatively and at the three-month postoperative timepoint. A minimal clinically important difference (MCID) occurred if a patient had an improvement greater than 12.8 for ODI and 1.2 for VAS back pain between preoperative and postoperative timepoints [16]. Moreover, to evaluate the accuracy of robotic assistance to place cortical screws, preoperative CT scans containing planned screw trajectories were overlaid with postoperative CT scans of placed screws using ExcelsiusGPS® software (Globus Medical, Inc., Audubon, PA, USA) to obtain mean tip, tail, and angular offset deviations.

Surgical technique

**Figure 1:**
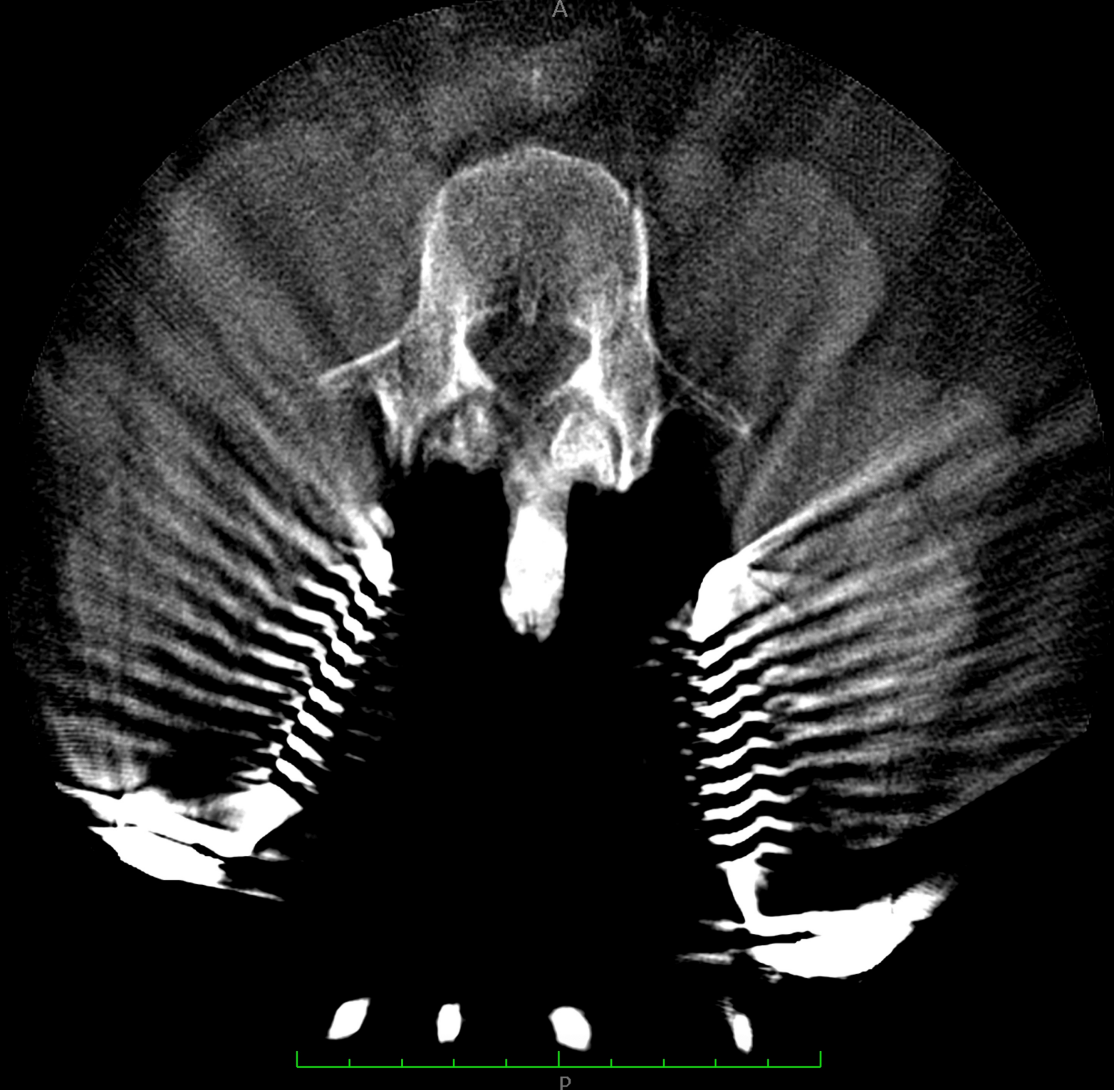
Preoperative 3D scan of patient #1. After the midline incision was made, the retractor was placed at the L3-S1 level.

**Figure 2:**
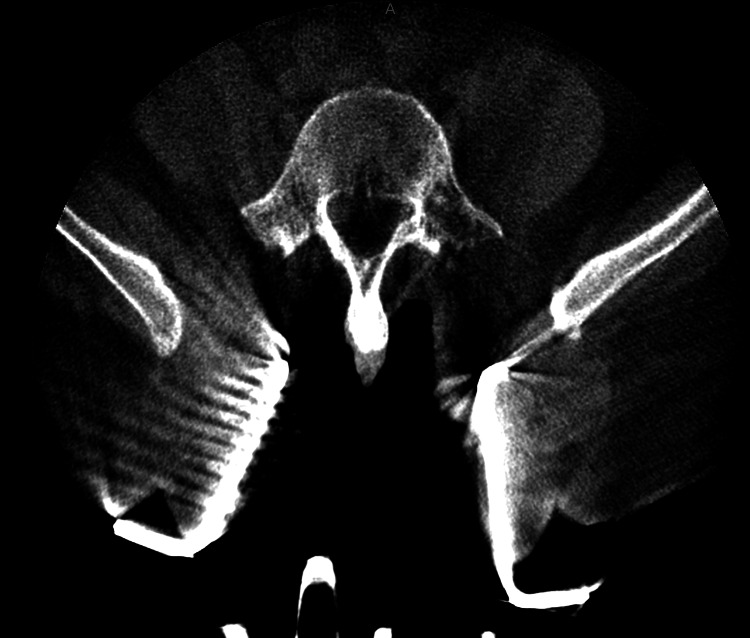
Preoperative 3D scan of patient #1. After the midline incision was made, the retractor was placed at the L3-S1 level.

**Figure 3:**
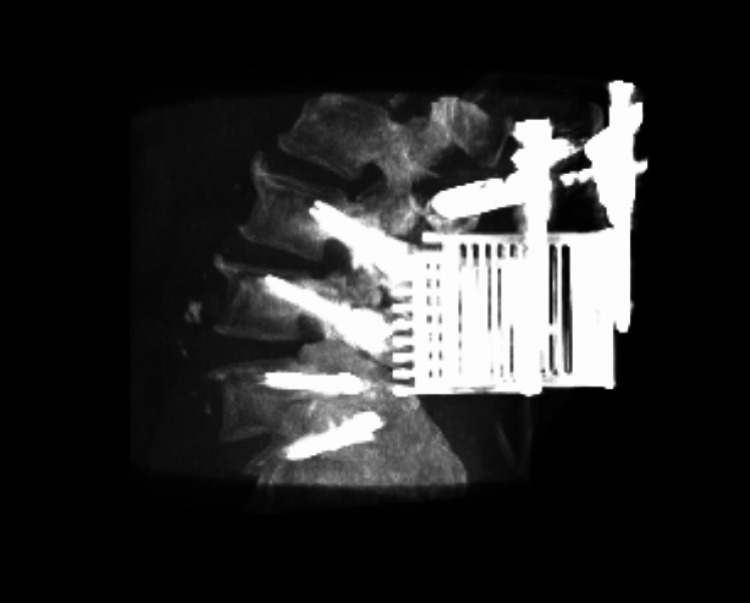
Postoperative 3D scan reconstruction of the L3-S1 MIDLIF approach with robotic assistance.

Preoperatively, CT scans of patients' lumbar spines were taken to plan cortical screw trajectory with robotic assistance (ExcelsiusGPS®, Globus Medical, Inc.); this is also referred to as pre-operative CT workflow. During surgery, the patient was placed under general anesthesia and laid in a prone position in the OR. Then, anteroposterior (AP) and lateral fluoroscopic images were taken and uploaded to the robotic system for registration with the preoperatively planned screw trajectories. To accurately track the real-time position of the patient's anatomy by the robot, a dynamic reference array (DRB) was placed on the left iliac crest, and a surveillance marker was placed on the right iliac crest. The navigated instruments’ tracking was then validated by robotic software, thus marking the end of registration. At the target levels, a midline incision was made, followed by dissection, retraction, and posterior direct decompression utilizing the technique described by Silva et al. in 2019. [7] Next, a thorough discectomy and end-plate preparation were undertaken, followed by the placement of an expandable spacer (RISE®, Globus Medical, Inc.) packed with autologous bone graft. Using navigated instruments and guidance from the robotic arm, cortical screws were placed based on the pre-planned trajectories. The final construct included posterior fixation with rods affixed to the cortical screws (Figures 1-3).

The top three comorbidities included hypertension (n=12), hypercholesterolemia (n=4), and diabetes (n=3). Cumulative diagnoses included spondylolisthesis (n=32), stenosis (n=24), and disc herniation (n=23). There were patients who had multiple comorbidities and diagnoses. Demographic information is included in Table 1.

**Table 1:** Demographics data

Total patients included (n)	42
Mean age (years)	53.6
Gender (%)	
Male	64.3
Female	38.1
Mean body mass index (BMI)	28.0
Comorbidities (%)	
None	57.1
Hypertension	28.6
Hypercholesterolemia	9.5
Diabetes	7.1
Other	2.3
Diagnosis (%)	
Spondylolisthesis	76.2
Stenosis	57.1
Disc herniation	54.8

Statistical analysis

Continuous interval data are presented as mean ± standard deviation, while categorical data are shown as percentages. The Shapiro-Wilk normality test was used to determine if continuous data were normally distributed. Paired t-tests with an alpha of 0.05 were used to determine the significance between preoperative and postoperative time points. All data analysis and visualization were performed using Microsoft Excel® 2017 (Microsoft, Redmond, WA).

## Results

The patient sample included a total of 42 patients (64.3% male) with an average age of 53.6 years and an average BMI of 28 kg/m². Twenty-five (25) (60%) patients underwent MIDLIF for single-level treatment, with the remaining patients receiving multi-level treatment. The cohort’s (n=42) mean operative time was 98.8 ± 12.7 mins, the mean estimated blood loss was 17.1 ± 11.4 mL, and the mean hospital length of stay was 3.4 ± 0.5 days. In single-level cases (n=25), the mean operative time was 98.8 ± 12.7 mins, the mean estimated blood loss was 17.1 ± 11.4 mL, and the mean hospital length of stay was 3.4 ± 0.5 days. In multi-level cases (n=17), the mean operative time was 98.9 ± 14.3 mins, the mean estimated blood loss was 18.6 ± 12.1 mL, and the mean hospital length of stay was 3.4 ± 0.6 days. There were no intraoperative complications reported. Complete intraoperative data are included in Tables 2, 3.

**Table 2:** Surgical data, raw data

Single-level	(# of patients)
L3-L4	1
L4-L5	21
L5-L1	3
Totals	25
Multi-level	(# of patients)
L2-L5	1
L3-L5	8
L3-L1	1
L4-L1	7
Totals	17

**Table 3:** Surgical data: analysis of the entire cohort (n=42).

	Average	Stdev
Operative time (mins)	98.8	12.7
Estimated blood loss (mL)	17.1	11.4
Hospital stay (days)	3.4	0.5
Single-level Cases (n=25)		
	Average	Stdev
Operative time (mins)	98.8	12.7
Estimated blood loss (mL)	17.1	11.4
Hospital stay (days)	3.4	0.5
Multi-level Cases (n=17)		
	Average	Stdev
Operative time (mins)	98.9	14.3
Estimated blood loss (mL)	18.6	12.1
Hospital stay (days)	3.4	0.6

Available radiographic data (n=20) demonstrated a low difference in deviation between the planned and placed cortical screw trajectories (mean tip deviation of 1.2 ± 0.6 mm, mean tail deviation of 1.1 ± 0.4 mm, and mean angular offset of 1.4 ± 0.7°). Complete screw accuracy measurements are included in Table 4. 

**Table 4:** Robots plan to place accuracy measurements on a per patient bias

	Tip Deviation (mm)	Tail Deviation (mm)	Angular Offset (°)
n	20	20	20
Mean	1.2	1.1	1.4
Standard deviation	0.6	0.4	0.7
Min	0.2	0.4	0.1
Max	2.1	2.3	3.0

Mean VAS back pain improved from 7.5 at baseline to 3.0 at three months postoperative. A similar trend was seen in mean ODI, which improved from 58.7 at baseline to 26.3 at the three-month postoperative visit. Mean VAS back pain and ODI were statistically significantly improved (p<0.05) between baseline and three months postoperative. Moreover, all patients (100%) achieved MCID for VAS back pain, and 98% of patients achieved MCID for ODI at the three-month postoperative visit. Clinical data are included in Table 5. 

**Table 5:** Patient reported outcomes VAS: Visual analogue scale, ODI: Oswestry disability index

	Pre-op	Three months post-op	p-value
n	42	42	
Mean VAS Back Pain	7.5 ± 0.7	3.0 ± 1.0	<0.0001
Mean ODI	58.7 ± 7.2	26.3 ± 10.3	<0.0001

Additionally, there were two postoperative complications reported at the three-month time point, including one wound infection and one wound dehiscence. The patient with a wound infection needed reoperation for debridement of the infection. For all complication information refer to Table 6.

**Table 6:** Complication data

Complications rates		
Intraoperative complication rate		0% (n = 0/42)
Postoperative complication rate		4.8% (n = 2/42)
Wound dehiscence		2.4% (n = 1/42)
Wound infection		2.4% (n = 1/42)
Reoperation rate		2.4% (n = 1/42)

## Discussion

In this paper, we present a cohort of 42 patients undergoing minimally invasive direct lateral interbody fusion (MIDLIF): operative metrics were remarkably consistent and favorable across both single- and multi‐level cases. The average operative time (~99 minutes), minimal blood loss (~17 mL), and brief hospital stay (~3.4 days) compare very favorably to published benchmarks for open and other minimally invasive lumbar fusion techniques, which often report longer operative times and greater transfusion rates. The absence of intraoperative complications further underscores the procedural safety of a lateral approach when combined with robotic planning and navigation.

Radiographic accuracy was excellent, with mean screw tip and tail deviations under 1.2 mm and angular offsets under 1.5°. These accuracy values meet or exceed thresholds generally associated with optimal clinical outcomes and reduced risk of neural or vascular injury. Such precision likely reflects the synergy between a minimally disruptive corridor and the robot-assisted trajectory planning, translating into consistent screw placement even in multi-level constructs.

Clinically, patients experienced statistically and clinically significant improvements in both back pain (VAS) and disability (ODI) by three months postoperatively. A 60% reduction in VAS (from 7.5 to 3.0) and over a 30-point drop in ODI mirror or surpass improvements reported in analogous studies at similar follow-up intervals. The 100% MCID achievement rate for pain and 98% for disability indicate nearly universal patient benefit especially notable outcomes given the known heterogeneity in pain response after lumbar fusion.

Supported by growing surgeon interest, MIDLIF is a mini-open posterior approach spinal surgery procedure developed to address some of the drawbacks of more well-established methods such as TLIF and PLIF. It requires less retraction of soft tissue and may reduce the risk of nerve root injuries due to the medial-to-lateral screw trajectory. A recent review of published clinical evidence shows MIDLIF to be a successful alternative to PLIF or TLIF, especially with respect to decreased blood loss, operative time, and length of stay, while achieving comparable clinical and radiologic outcomes [15-17]. However, there is a paucity of literature reporting outcomes of MIDLIF conducted with robotic assistance for the planning and placement of the cortical screws. To further build on this limited evidence base, this study evaluated a single surgeon's clinical experience in performing robotic-assisted MIDLIF with a focus on perioperative surgical outcomes, including screw plan-to-place accuracy, and early postoperative clinical outcomes.

Many surgeons have less experience using the medial-to-lateral cortical screw trajectory inherent to MIDLIF compared to traditional pedicle screw placement. Robotic assistance may be a valuable tool to overcome this experience gap and support the safe and reproducible placement of cortical screws. A major advantage of robotic assistance is the ability to pre-plan screw trajectories based on the patient's own CT scan.

Furthermore, the robotic arm guides the surgeon in placing the screws during surgery, enabling higher screw accuracy. A prior study with an alternative robotic system from that used in this study found that cortical trajectory screws could be placed at least as safely and accurately as those with CT-based navigation [18]. A previous clinical study by Jiang et al. used the same robotic system as our study and showed pedicle screw placement to be very accurate, with net linear deviation being 3.6 ± 2.3 mm and angular deviation being 3.6 ± 2.8° [19]. Our study has shown comparable accuracy results (Table 3) to Jiang et al. when placing cortical screws using the same robotic system, supporting the system’s versatility to place both pedicle and cortical screws in an accurate manner [19]. Moreover, no patients needed repositioning of screws, a complication commonly present in minimally invasive TLIF procedures [20].

Less anatomical disruption is characteristic of MIDLIF, and this may provide quicker recovery for patients and faster improvement in clinical outcomes. This study adds to the body of evidence supporting surgical outcomes with robotically assisted MIDLIF. Notably, the operative time and blood loss reported in the current study are lower than what has been reported for robotically assisted MIDLIF in a similar study out of the United States, while the length of stay was longer in the current series [15]. Differences may be attributable to various site-related factors, including the reported use of different robotic systems, surgical workflows, and country-specific healthcare considerations.

In addition to surgical outcomes, the current results enhance the robotically assisted MIDLIF literature through the inclusion of early postoperative clinical outcomes supporting the improvements achieved with this minimally disruptive procedure. Significant improvements in both back pain and disability at the three-month postoperative visit were observed, as compared to baseline. Additionally, 98% of patients achieved MCID for both VAS back pain and ODI. The mean three-month mean improvement in VAS back pain was 4.5 points, and ODI was 32.4 points, which is in line with 4.4 points and 26.4 points reported by other MIDLIF papers [21]. Moreover, the current results showed a low postoperative complication rate (4.8%), compared to the 6.9% and 7.6% seen in alternative methods like PLIF and TLIF [22]. Literature shows dural injury to be the most common intraoperative complication while using traditional methods [22]. There were no instances of dural injury in the current study, although the sample size is admittedly limited. The absence of dural injuries in this cohort may be partly ascribed to the robotically assisted screw placement accuracy. Another factor that may contribute to the low rate of dural and nerve injuries in the series is the use of expandable spacers, which allow for minimal cage size during insertion to reduce manipulation of the nerve roots.

Future studies may be desired to address some of the limitations of this single-center retrospective case series, including evaluating surgical and clinical outcomes across a wider number of surgeons and sites to ensure reproducibility and across an extended postoperative period to measure durability.

## Conclusions

Overall, this study illustrated that robotic-assisted MIDLIF can provide favorable clinical improvements to patients with degenerative disc conditions and allows for highly accurate placement of cortical screws while being safe for patients.

The robotic-assisted MIDLIF procedure makes use of robotic guidance for planning and accurate placement of cortical screws while needing minimal soft tissue retraction as compared to traditional methods. This single-center retrospective study showed robotic-assisted MIDLIF to be efficient, effective, and reproducible, with a low rate of related morbidity. Additionally, this study highlights that significant reductions in pain and disability can be seen as early as three months postoperatively, utilizing these less invasive techniques. And finally, the incorporation of robotic assistance enhances MIDLIF by yielding high cortical screw placement accuracy and low complication rates.
